# Occult hepatitis B in blood donation centers

**DOI:** 10.25122/jml-2023-0054

**Published:** 2023-04

**Authors:** Yazan AlRashdan, Khalid Al-Jaff, Manal Najdawi, Ala’ Sirhan

**Affiliations:** 1 Department of Pharmacy, Faculty of Pharmacy, Amman Arab University, Amman, Jordan; 2 Department of Applied Pharmaceutical Sciences and Clinical Pharmacy, Faculty of Pharmacy, Isra University, Amman, Jordan

**Keywords:** HBV markers, IgG, complement components (C3 and C4), serum ALP level

## Abstract

Occult hepatitis B (OHB) is characterized by the presence of hepatitis B virus (HBV) DNA in the blood of individuals who test negative for the hepatitis B surface antigen (HBsAg). OHB in blood donors can lead to HBV transmission through transfusions, yet the prevalence of OHB in Basrah, Iraq, is unknown. This study aimed to determine the prevalence of OHB in blood donation centers in Basrah and investigate the immune response to HBV in OHB-positive donors. We recruited 450 blood donors and categorized them into four groups based on HBV markers: the HBsAg-negative/HBsAb-negative/HBcAb-positive group, the recovery group (HBsAg-negative/HBsAb-positive/HBcAb-positive), the patient group (HBsAg-positive/HBsAb-negative/HBcAb-positive), and the apparently healthy group (negative for all HBV markers). We measured levels of IgG, IgM, complement components (C3 and C4), ALT, AST, and serum ALP in OHB-positive donors. Of the 450 donors, 97 (21.6%) were OHB-positive. IgG levels were significantly higher than IgM levels in OHB-positive donors. Healthy and HBsAg-negative/HBsAb-positive donors had significantly lower C3 levels than patients. IgG levels were significantly higher than IgM in both the patient and recovery groups. C3 levels were higher than C4 levels in all groups. The serum ALP level was significantly higher in the patient group. OHB prevalence in Basrah blood donors is high, indicating the potential for HBV transmission. OHB-positive donors showed an immune response to HBV. Our study provides insights into OHB prevalence and immune response in Basrah, with implications for diagnostic and therapeutic approaches in blood donation centers.

## INTRODUCTION

HBV is a serious global health concern that leads to liver cancer and cirrhosis. Roughly 40% of the world's population has either been exposed to or is a carrier of HBV, causing approximately one million HBV-related deaths annually [1]. HBV, which is highly species-specific, belongs to the Hepadnaviridae family and is a circular DNA virus that produces various protein products, including HBsAg, HBcAg, HBeAg, and DNA polymerase. These proteins are important for diagnosis and are measured through blood tests. Serological tests are essential for determining whether an HBV infection is acute or chronic, particularly in individuals with clinical symptoms or elevated ALT levels. Both molecular and serological testing methods are useful in determining a patient's HBV status [2]. However, it is critical to understand the relationship between the appearance of a marker and the patient's infection or disease status. The clinical usefulness of specific HBV markers has been clarified through HBV research, and their diagnostic use has been improved [3]. Blood is the primary vehicle for HBV transmission, and the safety of blood products is a significant issue in phlebotomy. Sensitive and specific HBV tests are crucial for defining the natural history of HBV infection and developing strategies to prevent transmission. Anti-HBc antibodies remain detectable for life and are markers of acute, chronic, or resolved HBV infection. They can be present in anyone who has been infected with HBV, even in the absence of both HBsAg and anti-HBs antibodies. Occult hepatitis B infection (OBI) refers to a form of hepatitis B characterized by the presence of HBV-DNA in the serum or liver of an infected individual, despite the absence of detectable HBsAg in their serum [4]. The underlying mechanisms responsible for the progression of OBI remain unclear, but some researchers suggest that low levels of HBV-DNA may lead to reduced HBsAg production, which remains below detectable levels [5]. In addition, genetic and immunological parameters may differ between resistant individuals and those with OBI [6, 7]. Anti-HBs antibodies are crucial for humoral immunity and play a critical role in protecting against potential HBV infections [8-10]. Despite the availability of sensitive screening assays for the detection of HBV, the disease remains a significant societal threat and is endemic in some medical institutions, with most cases being diagnosed in blood donation centers. Even with the availability of sensitive screening assays, there are occasional cases of post-transfusion HBV infections. As a result, individuals with OBI or those who are HBsAg-negative but HBcAb-positive may be unable to completely clear the virus and overcome the infection [10]. To identify and diagnose HBsAg-negative but HBcAb-positive individuals, we used an ELISA assay to screen for HBsAg and anti-HBc biomarkers in ostensibly healthy blood donors at the Central Blood Bank. We also aimed to detect potential OBI cases by identifying HBV-DNA in HBsAg-negative but HBcAb-positive donors, uncover cases of resolved infection, and assess relevant humoral immunity components to contribute to the understanding of the possible mechanisms responsible for the pathogenesis of OBI.

## Material and Methods

All procedures and data collection were conducted in compliance with the World Medical Association's Code of Ethics (Declaration of Helsinki). The study was approved by the ethics board committee of the Ministry of Health in Iraq.

### Subjects

The study was conducted between January 2017 and December 2018, with a total of 450 individuals of both sexes enrolled, including 192 patients, 128 apparently healthy individuals, and 130 HBsAg-negative but HBcAb-positive individuals.

### Collection of blood samples

A sterile plain tube with a volume of 10 ml was utilized to obtain a blood sample from the Basrah Blood Bank Center. After collection, the plasma was divided into numerous 250 µL aliquots and promptly stored at a temperature of -20°C for subsequent use. The plasma was subjected to various examinations, such as serological, molecular, and biochemical tests. Additionally, the humoral immunity components, such as Immunoglobulins (IgG and IgM) and complement C3 and C4 levels, were assessed.

### Serological tests by ELISA assay

#### Diagnostic kits used in the study

During the study, various ELISA kits were used, including the HBsAg ELISA kit from Plasmatec, USA; the HBcAb ELISA kit from Foresight, USA; the HBsAb ELISA kit from Dialab, Austria; the ExiprepTMplus viral DNA kit from Bioneer, Korea; the HBV PCR kit from Sacace, Italy; the C3 and C4 kit from LTA, Italy, and the IgM and IgG kit from LTA, Italy. The ELISA assay was carried out following the corresponding company protocol for each assay.

The blue color in the microtiter wells indicated a reactive result for HBsAg, while the absence of the color indicated a non-reactive result in the sample. The absorbance was measured at a wavelength of 450 nm. The cut-off value for the HBsAg marker was calculated using the following equation:

Cut-off value = 0.1 + NCx

NCx = Negative Control Mean absorbance

NCx = (NC1+ NC2)/2

Positive ≥0.5, Negative <0.1

Regarding HBcAb, the lack or minimal chromogenic response signifies its presence in the specimen. To quench the reaction responsible for the chromogenic transition from blue to yellow, a sulfuric acid solution was introduced into the microplate, and the threshold value for the anti-HBc biomarker was determined using the following equation:

Cut-off value = NCx*0.2

NCX = NC1 + NC2/2

Blank <0.05

Negative ≥1, Positive <0.08

To ascertain the concentration of the anti-HBs marker present in the specimens, a standard curve was constructed by plotting six standard concentrations (ng/ml) on the X-axis and their corresponding optical densities (OD) on the Y-axis. Our clinical assessment utilized the cut-off values of ≥10 gm/mL for a positive result and <10 gm/mL for a negative result.

### Molecular tests

#### Extraction of HBV DNA

**Figure 1:**
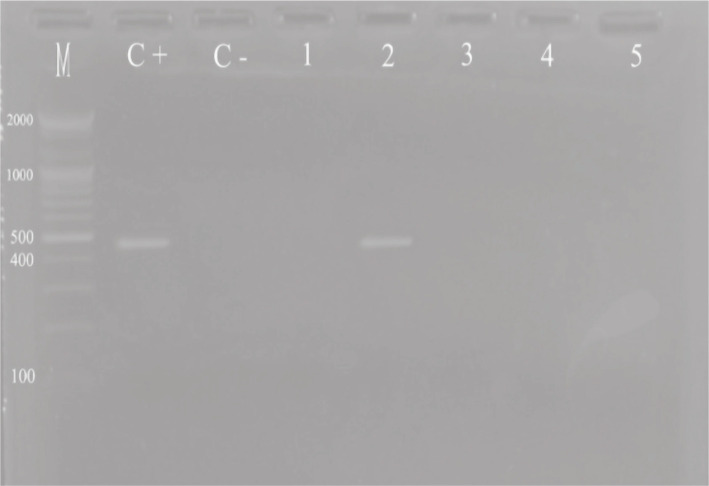
Electrophoresis of amplified HBV DNA Material.

The viral DNA was extracted from a 200 µl plasma sample using the Exprep™Plus Viral DNA/RNA Kits (Bioneer, Korea) in conjunction with an Automated Nucleic Acid Extraction System (Bioneer, Korea). Following completion of the protocol, the device door was opened to retrieve the elution and remove all remaining accessories from the base plate tube, after which the door was closed. The purified DNA was then subjected to amplification via the polymerase chain reaction (PCR) technique utilizing the HBV PCR kit (Sacace, Italy). The resulting amplified products were then separated via electrophoresis on 2% agarose gels, with the amplicon exhibiting a singular band and a length of 470 bp. Concurrent co-electrophoresis of the PCR ladder served to confirm the size of the amplicon. Visualization and photographic documentation of the DNA were achieved using a gel documentation system (as depicted in Figure 1).

### Humoral immunity tests

#### Determination of the IgG and IgM, and the C3 and C4 levels by the radial immunodiffusion plate

The concentration of immunoglobulins (IgG, IgM) and complement components (C3, C4) were measured via single radial immunodiffusion (SRID) using the protocol outlined in the LTA kit, Italy. The examined proteins were placed on agarose gel containing a specific antibody, forming an immune complex visible as a ring around the well. The diameter of the ring is directly proportional to the concentration of the analyzed protein, with the proportion corresponding to the diffusion time. A reference table was provided to establish the relation between concentration and procedure completion. Normal values for the IgM and IgG biomarkers in the kit were 60-280 mg/dl and 800-1800 mg/dl, respectively, while normal values for the C3 and C4 biomarkers were 91-156 mg/dl and 20-50 mg/dl, respectively.

### Biochemical tests

The Abbott C4000 system (Architect plus, USA) was employed. The system was activated, and the liver function test feature was selected using the touch LCD screen to initiate the test. The system carrier was then installed in the system handle to start the test, with the resultant output retrieved using the system primer. The normal values for BiliD in the kit were 0.0-0.5 mg/dl, for BiliT 0.2-1.2 mg/dl, ALT 0-55 U/L, AST 5-34 U/L, and for ALP 40-150 U/L.

### Statistical analysis

Statistical analysis was conducted using the Statistical Package for Social Sciences (SPSS) version 15 software. Mean values and standard deviations (M±SD) were calculated for humoral immunity and biochemical parameters. Differences in mean values for variables in more than two groups were assessed using the ANOVA - LSD test, with a p-value of less than 0.05 considered statistically significant.

## Results

### Characterization of the study population

A total of 450 blood donors, including males and females, were recruited and divided into four groups for the study. The first group consisted of apparently healthy individuals, with 128 male participants between the ages of 21 and 46. The second group, consisting of 130 patients, included 127 males and 3 females with ages ranging from 20 to 50. The third group, comprising 192 participants, was divided into two subgroups based on serological status. The HBsAg-negative/HBcAb-positive group had 109 participants, while the second subgroup had 83. There were 186 males and 6 females with ages ranging from 18 to 59 years.

### Prevalence of HBsAg−/anti-HBc+

According to the annual report issued by the blood bank center, between Jan. 2015 and Dec. 2016, a total of 144,838 individuals donated blood. The report indicated that the prevalence of anti-HBc, either with or without HBsAg, was 3358 (2.32%), HBsAg (with or without anti-HBc) was 295 (0.21%), anti-HCV+ was 160 (0.11%), anti-HIV+ was 0 (0%), and VDRL was 610 (0.42%). Out of the 4324 infected persons, 3358 (77.66%) had anti-HBc (with or without HBsAg), 295 (6.82%) had HBsAg (with or without anti-HBc), 160 (3.7%) had anti-HCV+, and 610 (14.1%) had VDRL. The prevalence of HBsAg−/anti-HBc+ and HBsAg+/anti-HBc− among the infected persons was 3259 (75.37%) and 196 (4.53 %), respectively.

### Serological tests

The screening ELISA tests of all 450-blood donors revealed that they were divided into four groups based on the presence of HBsAg, HBsAb, anti-HBc antibodies, and HB viral DNA. The HBsAg- /HBcAb+ group comprised 192 (42.67%) individuals and was divided into two subgroups: the first subgroup, HBsAg−/anti-HBc+, consisted of 83 patients (18.44%) in recovery, and the second subgroup comprised 109 patients (24.22%) who were positive for HBsAb. The third group included 130 patients (28.89%) who were positive for HBsAg and HBcAb but negative for HBsAb. The fourth group comprised 128 healthy individuals (28.44%) who tested negative for all HBV markers (Table 1).

**Table 1:** Serological and molecular tests.

Study groups	HBsAg	HBsAb	Anti-HBcAb	HBV-DNA
**HBsAg-/anti-HBc+ n=83 (18.44%)**	-	-	+	-
**Recovery n=109 (24.22%)**	-	+	+	-
**Patients n=130 (28.89%)**	+	-	+	+
**Healthy n=128 (28.44%)**	-	-	-	-

#### Molecular tests

The detection of HBV-DNA was limited to the patient group and the positive control, and no traces of it were found in any of the other groups that were examined, including the HBsAg-/HBcAb+ group, healthy individuals, and the negative control (as depicted in Figure 1).

### Humoral immunity tests

#### Serum levels of IgG and IgM

According to the results of the study, the patient group exhibited significantly higher IgG levels (mean±SD mg/dL) compared to the other groups studied (P < 0.001), with a mean value of 1382.06±891.82. The recovery group also showed significantly elevated IgG levels (P < 0.05) with a mean value of 954.67±336.55. However, there were no significant differences in the IgM levels among all the groups, as shown in Table 2.

**Table 2:** Humoral immunity tests (IgM and IgG mg/dl).

Study groups (mg/dl)	IgM (Mean±SD) Minimum–Maximum	IgG (Mean±SD) Minimum–Maximum
**HBsAg−/anti-HBc+**	817.9±407.3 (173.7–1636.4)	738.797±416.66 (162.5–1628.3)
**Recovery**	730.16±439.02 (162.7–1618.4)	954.67±336.55 (437.5–1728.4)*
**Patients**	820.23±340.4 (437.5–1728.4)	1382.06±891.82 (537.5–3368.8)**
**Healthy**	769.34±439.62 (142.3–1328.1)	844.65±490.57 (162.7–1628.4)

* – P<0.05; ** – P<0.001; normal values for IgG: 800–1800 mg/dl, and IgM: 60-280 mg/dl.

#### Serum levels of complements components (C3 and C4)

The patient group had significantly higher C3 levels (mean±SD mg/dl) compared to the healthy group and HBsAg−/anti-HBc+ group (P<0.05), with a mean value of 101.93±33.06. On the other hand, the C3 levels of the healthy group and HBsAg−/anti-HBc+ group were significantly lower, with mean values of 70.78±22.69 and 72.09±28.49, respectively. However, there was no significant difference in C4 levels among all groups, as presented in Table 3.

**Table 3:** C3 and C4 serum levels (mg/dl).

Study groups (mg/dl)	C3 Mean±SD (Minimum–Maximum )	C4 Mean±SD (Minimum–Maximum )
**HBsAg−/anti-HBc+**	72.09±28.49 (39.1–84.4)*	19.51±13.12 (5–52.7)
**Recovery**	89.91±27.81 (44.4–141.5)	17.84±7.54 (5–37.8)
**Patients**	101.93±33.06 (68–186.1)	23.28±14.81 (5–52.7)
**Healthy**	70.78±22.69 (39.1–102.1)*	22.09±4.269 (4–52.7)

Normal values for C3=91–156 mg/dl, C4=20–50 mg/dl; *– P<0.05.

### Biochemical tests

The patient group showed significantly higher levels of direct serum bilirubin 0.21±0.15 than the recovery group 0.13±0.06, with a statistical significance of P<0.01. Moreover, the patient group displayed significantly elevated levels of indirect bilirubin 0.66±0.42 compared to the other groups, with a statistical significance of P<0.001, as depicted in Table 4. It is noteworthy that jaundice is a common symptom in hepatitis A, B, and C infections, with the prevalence ranging from 20% to 70%, depending on the virus. No significant differences were found in the levels of ALT and AST among groups (Tables 5 and 6). The serum ALP level in the patients' group (70.6±21.05) was significantly higher (P<0.01) than that in the recovery group (56.5±15.18). It is important to note that although the serum ALP levels were elevated, they remained within the normal range.

**Table 4:** Serum bilirubin levels (mg/dl).

Study groups	Direct Bilirubin (mg/dl) Mean±SD(Minimum–Maximum)	Indirect Bilirubin (mg/dl) Mean±SD(Minimum–Maximum)
**HBsAg−/anti-HBc+**	0.14±0.06 (0.1–0.3)	0.36±0.15 (0.2–0.7)
**Recovery**	0.13±0.06 (0.1–0.3)	0.36±0.16 (0.1–0.9)
**Patients**	0.21±0.15 (0.1–0.7)*	0.66±0.42 (0.2–1.7)**
**Healthy**	0.20±0.11 (0.1–0.4)	0.73±0.14 (0.1–0.8)

Normal values for direct bilirubin (0.0–0.5 mg/dl), for indirect bilirubin (0.2-1.2 mg/dl); * – P<0.05; **P<0.001.

**Table 5:** ALT (IU/L) and AST (IU/L) serum levels.

Study groups	ALT Level (IU/L) Mean±SD(MinimumMaximum)	AST Level (IU/L) Mean±SD(MinimumMaximum)
**HBsAg−/anti-HBc+**	16.2±8.89 (6–37)	18.5±6.62 (9–35)
**Recovery**	15.2±7.56 (7–36)	19.36±7.77 (8–40)
**Patients**	21.4±9.57 (7–51)	21.86±7.53 (13–37)
**Healthy**	18.6±7.79 (5–29)	17.3±8.62 (8–37)

Normal values for ALT: 0–55 IU/L, AST:5–34 IU/L.

**Table 6:** ALP serum levels (IU/L).

Study groups	Mean±SD (Minimum–Maximum)
**HBsAg−/anti-HBc+**	59.45±19.06 (39–90)
**Recovery**	56.5±15.18 (22–91)
**Patients**	70.6±21.05 (35–104)*
**Healthy**	57±16.9 (28–85)

Normal value for ALP: 40–150 IU/L; * – P<0.001.

## Discussion

### Characterization of the study population

The study enrolled 450 blood donors, with a majority of 98% (441) being males and only 2% (9) being females. This gender distribution in the sample is attributed to the higher likelihood of males donating blood due to their higher levels of activity in the community, which puts them at a higher risk of acquiring or transmitting an HBV infection. The mean age of the blood donors was 29 years, with a standard deviation of 6 years.

### Prevalence of HBsAg−/anti-HBc+

The annual report from January 2018 to December 2019 identified Basrah city as a high-risk area for HBV infection, with a previous study showing that the virus is endemic in some medical institutions in the area [11]. HBV infection prevalence differs across regions and is shaped by human behavior and socio-cultural practices, leading to different epidemiological patterns [12]. The number of patients with HBV in Basrah has risen significantly, with cases frequently detected incidentally during blood transfusions or indirect tests in several medical facilities, including the hemodialysis unit, oncology unit, blood bank center, department of public health, and Center for Hereditary Blood Diseases in the Basrah governorate.

### Serological tests

The study analyzed the results of 450 blood donors, divided into four groups based on their ELISA results for HBsAg, HBsAb, anti-HBc antibodies, and HB viral DNA (Table 1). These diagnostic parameters are critical for accurately diagnosing the disease and identifying cases under investigation. Previous research suggests that HBV replication is not efficient immediately after infection, and HBV-DNA and antigens remain undetectable until 4 to 7 weeks post-infection. Studies have demonstrated that early immunological responses are not activated by HBV until the delayed exponential phase of replication begins [13]. The use of HBsAg alone as a marker for detecting HB virus in medical institutions was found to be inadequate.

On the other hand, HBcAg is a unique immunogen that serves as both a T cell-independent and a T cell-dependent antigen in both mice and humans. It is approximately 1000 times more immunogenic than HBeAg. The spike region of HBcAg is responsible for binding naive B cells, which then act as efficient antigen-presenting cells of HBcAg to the T helper cells [14]. Studies have reported that T cell response to HBcAg is associated with the resolution and seroconversion of chronic hepatitis B. Although anti-HBc IgG can remain positive for many years after exposure to HBV, it is not a protective antibody [15].

The investigation identified 109 individuals who fully recovered from acute infections by forming anti-HBs. During the acute stage, detection of anti-HBc in the HBsAg-/anti-HBc+ group can lead to false positive results, and the patient may be considered non-immune. Alternatively, patients may have had resolved infection within an anti-HBc window, which leads to incomplete recovery cases with gradually decreasing anti-HBs titers. In most chronic infection cases, HBsAg and HBV-DNA are detectable [16]. Antibody production during acute hepatitis plays a vital role in neutralizing free HBV particles, and inhibiting viral entry into host cells, thus limiting the cell-to-cell spread of viral particles. The binding of anti-envelope antibodies with an excess of viral envelope antigens produced during virus replication may lead to the inability to detect these antibodies. The outcomes of acute HBV infection are linked with various adaptive immune response profiles. A self-limited resolution shows a robust, multispecific antiviral CD4+ and CD8+ response, along with sufficient anti-envelope antibodies. According to estimates, the HBsAg clearance rate in Western patients is less than 2%, whereas, in patients of Asian origin, it is even lower, ranging from 0.1% to 0.8%. The complete mechanism of occult hepatitis B virus (HBV) infection remains largely unclear.

Nevertheless, research has suggested that those with this type of infection cannot detect hepatitis B surface antigen (HBsAg) and HBV-DNA in their blood [5]. Often, patients only have anti-HBc, an antibody produced against the core antigen of HBV, as an indication of HBV infection. Confirming a diagnosis of chronic HBV infection with an HBsAg-negative variant requires assessing the HBV-DNA level, and a positive test result can effectively support the diagnosis. Despite the current vaccination programs and the continuous attempts to reduce HBV transmission, the virus remains a significant public health problem. The risk of HBV infection among healthcare workers (HCWs) is one of the major concerns in the health sector. The prevalence of HBV infection among HCWs has been reported to be higher than in the general population in several countries [17-19].

The risk of occupational exposure to HBV is high, and the transmission of HBV in healthcare settings is well-documented [20]. A study conducted in Saudi Arabia revealed that the prevalence of HBV among HCWs was 2.9% [21]. The most significant risk factors for HBV infection among HCWs were identified as the number of years in practice, not wearing protective clothing, and the number of previous needle stick injuries. Therefore, it is necessary to establish effective preventive measures and vaccination programs for HCWs to reduce the risk of infection in healthcare settings.

Consequently, our findings suggest that using a combination of markers, such as HBsAg, HBsAb, anti-HBc antibodies, and HB viral DNA, is necessary to accurately diagnose and treat HBV infection. Moreover, understanding the immune response profiles in different infection stages can help predict the outcomes of acute HBV infection. It is also essential to establish effective preventive measures and vaccination programs for HCWs to reduce the risk of infection in healthcare settings.

### Molecular tests

The presence of anti-HBc antibodies in HBsAg-negative chronic hepatitis B patients can increase the risk of cirrhosis or hepatocellular carcinoma (HCC) as HBV-DNA can still be detected by PCR assay in serum and liver [2]. However, the levels of HBV-DNA are usually low. It is well-documented in the scientific literature that HBV transmission from donors with anti-HBc antibodies can occur, particularly in settings where there is a potential transfer of substantial quantities of the virus to susceptible individuals, such as liver transplantation or blood transfusion [22, 23]. Individuals who test positive for anti-HBc antibodies usually have a low risk of transmitting HBV, [24], except in high-risk environments [25]. To reduce the risk of transmitting or reactivating HBV through blood transfusions, it is recommended to administer hepatitis B immune globulin before surgery and provide prophylactic antiviral therapy after the procedure [26]. It is difficult to eliminate the covalently closed circular form of HBV-DNA (cccDNA), and HBV surface antigen clearance is rare even after a year of treatment [27].

### Humoral immunity tests

#### Serum levels of IgG and C4

In adults, HBV infection typically results in an acute, self-limiting infection that triggers a broad and effective immune response. However, in chronic hepatitis B patients, the immune system fails to efficiently recognize and control the virus, leading to chronic liver inflammation that can eventually result in cirrhosis and hepatocellular carcinoma (HCC) [28]. The initial defense against HBV infection is believed to be mediated by nonspecific mechanisms, such as killing virus-infected cells by natural killer (NK) cells and the secretion of antiviral cytokines. However, the induction of virus-specific immunity requires several days, during which the virus can replicate unchecked [29]. Upon initial infection, IgM is the first antibody in the serum and mucosa, neutralizing free viruses and triggering the classical complement pathway [30]. IgM is present in lower concentrations compared to IgG and IgA [31],; however, it is more effective in activating the complement pathway and performs essential functions such as agglutination, opsonization, and toxin neutralization. Additionally, IgM acts as a surface receptor for antigens on B cells.

Subsequently, IgG is produced sequentially after IgM and provides long-term protection against viral infections [32]. IgG is the most abundant antibody class produced by plasma cells derived from B cells that have undergone class switching. While IgM antibodies are dominant in the early stages of the antibody response, IgG and IgA become the predominant antibody classes later, with IgE contributing to a minor part of the response. IgG antibodies protect against bacteria and viruses, neutralize toxins, and squeeze between cells to eliminate bacteria and other invaders infiltrating the cells or skin. Antibodies are proteins produced by B cells in response to a foreign antigen, such as a virus or bacteria. They can recognize and bind to specific antigens on the surface of the virus, neutralizing it and preventing it from infecting host cells. In the case of HBV, antibodies are critical for controlling the infection and clearing the virus from the body. There are several types of antibodies that can be produced against HBV, including IgM and IgG. IgM is the first antibody produced in response to an infection and is important for neutralizing free virus particles in the early stages of infection. IgG is produced later in the immune response and provides long-term protection against the virus. However, some individuals may not produce enough antibodies against HBV, which can result in chronic infection [33].

In some cases, this may be due to genetic factors, such as polymorphisms in HLA class I and II genes. Other factors that can affect antibody production include age, health status, and vaccination history. Overall, humoral immunity is an important component of the immune response against HBV, and the production of antibodies is critical for controlling the infection and preventing the development of chronic liver disease.

#### Serum levels of complements components (C3 and C4)

The complement system also has important roles beyond direct involvement in the immune response to viral infections. It is involved in the clearance of immune complexes and apoptotic cells and plays a role in tissue repair and regeneration [34]. Complement activation can also contribute to inflammation, and excessive complement activation has been implicated in a variety of pathological conditions, including autoimmune diseases, neurodegenerative disorders, and ischemia-reperfusion injury [35]. In the context of viral hepatitis, there is evidence that complement activation may contribute to liver injury and fibrosis. In addition, the complement system may play a role in the pathogenesis of extrahepatic manifestations of HBV infection, such as membranous glomerulonephritis and vasculitis [36].

Further research is needed to fully understand the complex interplay between the complement system and HBV infection and to develop strategies for modulating complement activation as a therapeutic approach to viral hepatitis and related complications. The study investigated the humoral immune response in patients with hepatitis B surface antigen-negative/hepatitis B core antibody-positive (HBsAg−/anti-HBc+) status, a population known to harbor occult hepatitis B virus (HBV) infection. The results revealed that the level of immunoglobulin G (IgG) was significantly reduced in the HBsAg−/anti-HBc+ group compared to other studied groups, indicating a potential defect in IgG production or generation of specific IgG against HBsAg. Notably, the levels of IgG were significantly higher in the HBsAg−/anti-HBc+ group compared to healthy controls. Interestingly, the study showed that patients and recovery groups had significantly higher levels of IgG than IgM, implying a shift from early-phase humoral immunity to the later phase of antibody response.

Furthermore, the levels of complement component C3 were much higher than those of C4 among groups, suggesting the crucial role of C3 in the classical, alternative, and lectin pathways of complement activation. The findings are consistent with previous reports that chronic HBV patients have significantly higher levels of IgA, IgG, and IgM, while the serum C3 and C4 levels were significantly lower compared to healthy controls [37]. Additionally, another study found that occult HBV infection patients had significantly lower serum levels of IgG and C4, while IgM and C3 were higher than healthy controls [38]. The study provides evidence that HBsAg−/anti-HBc+ patients can mount a humoral immune response, but specific defects may exist in the production of IgG or generation of specific IgG against HBsAg. Further research is warranted to elucidate the underlying mechanisms and potential therapeutic interventions for occult HBV infection.

### Biochemical tests

Since HBV infection does not directly damage hepatocytes, the control of viral spread is mainly achieved by the action of HBV-specific CD8+ T cells, which eliminate infected hepatocytes through cytolysis. Consequently, it has been proposed that altered levels of alanine aminotransferase (ALT), a marker of liver injury, may reflect the presence or absence of HBV-specific T cells [35]. The elevation of ALT levels in HBV infection could be due to increased activities of the T-cell response, which distracts and kills infected hepatocytes, or to a lack of HBeAg, which may lead to low T-cell response and T-cell deletion [35]. Therefore, normal ALT levels in the majority of HBV-infected patients suggest a lack of HBV-specific T-cell response. However, the adoptive transfer of HBV-specific T cells has been shown to cause substantial inhibition of HBV replication without any increase in ALT levels, which suggests that cytokine-mediated non-cytopathic virus control is also involved in virus clearance. Additionally, quantifying HBV-specific T cells in the blood and liver does not always correlate with ALT levels [13]. Instead, liver inflammation, associated with elevated ALT levels, is characterized by the infiltration of inflammatory cells, such as granulocytes, monocytes, and non-antigen-specific T cells [36].

## Conclusion

This study is the first to evaluate the humoral immunity factors in patients with chronic hepatitis B virus (HBV) infection who are negative for HBV surface antigen (HBsAg) but positive for antibody to HBV core antigen (anti-HBc). The results suggest that patients can initiate humoral immune responses but have a defect in generating specific IgG against HBsAg. This finding could explain why some patients are unable to clear HBV infections despite the presence of other humoral immune factors. These results provide new insights into the mechanisms underlying chronic HBV infection and may have implications for developing new therapeutic approaches.

## Personal thanks

We would like to thank the blood bank personnel in Basrah, Iraq, for their invaluable contribution towards successfully completing this project. Their support has been exemplary and has enabled us to achieve our research objectives with utmost diligence and meticulousness.
